# Relationships of high mean corpuscular volume, low total iron binding capacity and protein-energy wasting in patients on maintenance hemodialysis

**DOI:** 10.1007/s10157-026-02887-w

**Published:** 2026-06-02

**Authors:** Misa Ikeda-Taniguchi, Keiichi Hirao, Keigo Shibagaki, Hirokazu Honda

**Affiliations:** 1 Department of Nephrology, Showa Medical University Graduate School of Medicine, 1-5-8 Hatanodai, Shinagawa Ward, Tokyo, 142-8666 Japan; 2 Shibagaki Dialysis Clinic Togoshi, Tokyo, Japan; 3 Shibagaki Dialysis Clinic Jiyugaoka, Tokyo, Japan

**Keywords:** Functional iron deficiency, Hemodialysis, Mean corpuscular volume, Protein-energy wasting, Total iron binding capacity

## Abstract

**Background:**

Recent studies have reported that high mean corpuscular volume (MCV) may be associated with mortality in chronic kidney disease patients. A high MCV is caused by erythropoietin-stimulating agent (ESA). Most patients with protein-energy wasting (PEW) have functional iron deficiency (FID) and need a high ESA dose. FID is associated with low total iron binding capacity (TIBC), which is a marker of iron status, malnutrition, and inflammation, and it may be a predictor of muscle loss. The relationships between high MCV and iron status markers in patients on hemodialysis (HD) are not well known. The aim of this study was to assess the relationships between high MCV and iron status markers in a cross-sectional and longitudinal study considering nutritional status, inflammation, muscle mass, and hydration status.

**Methods:**

A prospective cohort of 39 HD patients was examined for 12 months. The relationships between MCV and iron status markers (TIBC, ferritin, hepcidin-25, iron) were examined using multivariate linear regression analysis and multivariate repeated measures analysis.

**Results:**

At baseline, a high MCV (≥ 100 fL) was significantly related to low TIBC and low muscle mass. Multiple regression analysis showed that MCV was associated with low TIBC. On multivariate repeated measures analysis, MCV was associated with a sustained low TIBC, and proportion of PEW and ERI were associated with a sustained high MCV.

**Conclusion:**

High MCV was associated with low TIBC. Furthermore, a sustained high MCV suggested ESA hypo-responsiveness and tendency to iron use disorder and may be a useful marker of PEW.

**Supplementary Information:**

The online version contains supplementary material available at 10.1007/s10157-026-02887-w.

## Introduction

Recent studies reported that a high mean corpuscular volume (MCV) may be associated with mortality in chronic kidney disease (CKD) patients. It is reported that 5% up to 30% end-stage kidney disease patients have macrocytosis [1–3]. A high MCV is caused by vitamin B12 or folate deficiency, hypothyroidism, alcoholism, liver disease, dialysis-related changes in erythrocytes, ferrotherapy, and a high erythropoietin-stimulating agent (ESA) dose [1–5]. Hsieh et al. reported that a higher MCV was associated with nutritional markers. Crystal osmotic pressure has been reported to be an important regulator of erythrocyte volume, and a reduced crystal osmotic pressure as a result of malnutrition may cause erythrocyte swelling and a higher MCV [2].

Protein-energy wasting (PEW) is common and characteristic of malnutrition in hemodialysis (HD) patients. It is characterized by decreased skeletal muscle mass and plasma protein and is associated with mortality [6, 7]. Most patients with PEW have functional iron deficiency (FID) and need a higher ESA dose [8–12]. FID is associated with low total iron binding capacity (TIBC). TIBC is a marker of iron status, malnutrition, and inflammation, and it may be a predictor of muscle loss [8–10]. From these relationships, a high MCV caused by a high ESA dose suggests FID and malnutrition.

Based on previous studies, we hypothesized that HD patients with a high MCV have low TIBC and FID. The relationships between a high MCV and iron markers in HD patients are not well known. The aim of this study was to assess the relationships between a high MCV and iron status markers in a cross-sectional and longitudinal study considering nutritional status, inflammation, muscle mass, and hydration status. We also assessed the relationship between high MCV and PEW.

## Materials and methods

### Study design, setting, and participants

A prospective cohort of 39 HD patients was examined for 12 months at two clinics. Exclusion criteria were: absolute iron deficiency defined by the guideline for treatment of anemia from the Japanese Society of Dialysis Therapy [13], malignancy, infection, acute vasculitis, active hepatitis, liver failure, heart failure (NYHA class II to IV), taking corticosteroids, and hospitalized during the observation period. Medical treatments used to manage the conditions of all patients were similar, and the medications for each patient were prescribed according to the K/DOQI guidelines [14]. This study complied with the Declaration of Helsinki. Ethical approval was obtained from the ethics committee of Showa Medical University (1259). Informed consent was obtained from all individual participants included in the study.

### Measurement items

Red blood cell (RBC), mean corpuscular volume (MCV), mean corpuscular hemoglobin concentration (MCHC), creatinine, urea, albumin, iron (Fe), TIBC, ferritin, hepcidin-25, and C-reactive protein (CRP) levels were measured using routine procedures. Iron status markers, erythrocyte indices (RBC, MCV, MCHC), and albumin were measured every 3 months.

### Nutritional status

Nutritional status was assessed by the serum albumin level and the normalized protein catabolic rate (nPCR) every 3 months. Patients with PEW were assessed according to the International Society of Renal Nutrition and Metabolism (ISRNM) criteria to determine if they have PEW [6]. In this study, we defined patients with risk for PEW as “pre-PEW”. Their nutritional status was matched of two out of four items of the ISRNM criteria.

### Hydration status and muscle mass

Hydration status was evaluated using the extracellular body water/total body water (ECW/TBW) ratio [14, 15]. TBW at baseline, 3, 6, 9, and 12 months was calculated using the Watson formula, as follows [16–18]:

Males: 2.447—(0.09156 × age) + (0.1074 × height) + (0.3362 × postdialysis body weight).

Females: – 2.097 + (0.1069 × height) + (0.2466 × postdialysis body weight).

ECW was estimated by the Peter formula, as follows [18]:

Males: – 2.47 × 0.842 + 8.76 × body surface area.

Females: – 1.96 × 0.527 + 8.05 × body surface area.

Body surface area: postdialysis body weight 0.5378 × height 0.3964 × 0.024265.

Intracellular water (ICW) was calculated by subtracting ECW from TBW.

Muscle mass was evaluated using creatinine (Cr) and ICW [19]. EBW/TBW and ICW were assessed every 3 months.

### Treatment of renal anemia

Renal anemia was treated with darbepoetin alfa and methoxy polyethylene glycol-epoetin beta. Anemia treatment was managed according to the Japanese dialysis guideline, and each primary physician managed the doses for ESA therapy and ferrotherapy [13].

To transform ESA doses to units of epoetin alfa, we used conversion factors of 250:1 for darbepoetin alfa and 208:1 for methoxy polyethylene glycol-epoetin beta [20]. The ESA-resistance index (ERI) was calculated as ESA dose (epoetin alpha units/week) divided by (hemoglobin [g/dl] × body weight after HD [kg]) at baseline.

### Statistical analyses

Data are presented as mean ± standard deviation values or median (range) values unless otherwise noted, and P values < 0.05 were considered significant. Comparisons between two groups of normally distributed variables were performed using Student’s *t*-test, and the Wilcoxon rank-sum test was used for non-normally distributed variables. For nominal variables, Fisher’s exact test was used for comparisons between two groups.

Baseline data were stratified into two groups by MCV size. A high MCV was defined as being 100 fL or more. Multiple linear regression analysis was performed to assess the associations between TIBC as a dependent variable and interactions of MCV with markers of iron status (log ferritin, log hepcidin-25) or inflammation markers (log CRP) as independent variables at baseline or malnutrition or muscle mass (Cr, ICW) or hydration status (ECW/TBW).

Statistical analysis was performed to investigate the relationships with changes of iron status markers and MCV using two-way ANOVA. The effects on TIBC by MCV were evaluated using odds ratios (ORs; 95% confidence intervals [CIs]). Associations between variables of TIBC estimated by more than two equal time points and MCV were assessed by multivariate repeated measures analysis.

Data were analyzed using JMP Student Edition version 18.0.1 (SAS Institute, Cary, NC) and Prism version 7.0 (GraphPad Software, Inc., La Jolla, CA).

## Results

### Patients’ characteristics and biomarkers at baseline by MCV size

A total of 39 patients were analyzed. The patients were stratified into two groups by MCV size (Table 1). High MCV (MCV ≥ 100 fL) was not significantly associated with age, gender, HD vintage, Kt/V, albumin, nPCR, CRP, ERI, proportion of ferrotherapy, and ECW/TBW. TIBC, DW, Cr, and ICW were significantly lower with a high MCV. Proportion of PEW and pre-PEW, and proportion of ESA users were significantly higher with a high MCV.

**Table 1:** Patient characteristics and laboratory data at baseline in all patients and patients grouped according to MCV size

	All (N = 39)	MCV < 100 fl (N = 27, 69%)	MCV*≥*100fl (N = 12, 31%)	P
Age (year)	69 (41, 87)	68 (41, 78) *	71 (43, 87)	0.38
Gender (% of men)	59	59	58	0.96
**Dry weight (kg)**	**53.5 (37.3, 75.2)**	**57.6 ± 2.0 **^**†**^	**46.8 ± 2.9**	**0.0079**
BMI (kg/m^2^)	20.3 (15.3, 26.9)	20.5 (15.3, 26.9)	19.2 (15.9, 22.6)	0.060
BMI < 18.5 (%)	24.2	17.4	40.0	0.16
Dialysis vintage (months)	127 (25, 320)	137 (31, 320)	97 (25, 253)	0.48
DM (%)	17.1	21.7	8.3	0.32
Kt/V	1.48 ± 0.18	1.45 ± 0.035	1.54 ± 0.056	0.22
nPCR (g/kg/day)	1.1 (0.71, 1.4)	1.1 (0.71, 1.4)	0.94 (0.77, 1.2)	0.17
**Proportion of PEW and pre-PEW (%)**	**11.4**	**0.0**	**40.0**	**0.0007**
Hb (g/dL)	10.9 ± 0.89	10.9 (9.5, 12.8)	11.3 (9.0, 12.4)	0.50
**MCV (fL)**	**96.3 ± 6.8**	**93.2 ± 0.97**	**103.1 ± 1.5**	** < 0.0001**
MCHC (%)	32.0 ± 0.96	32.1 (30.2, 33.8)	32.3 (29.4, 33.1)	0.70
**Creatinine (mg/dL)**	**11.8 ± 2.7**	**12.4 ± 0.50**	**10.3 ± 0.73**	**0.043**
Albumin (g/dL)	3.9 ± 0.30	3.9 (3.5, 4.4)	4.0 (3.2, 4.3)	0.42
Albumin < 3.8 g/dL (%)	22.9	16.0	40.0	0.14
CRP (mg/dL)	0.050 (0.011, 0.89)	0.050 (0.013, 0.73)	0.052 (0.011, 0.89)	0.50
Iron (μg/dL)	71 ± 21	68 (24, 115)	71 (43, 121)	0.71
**Total iron binding capacity** **(μg/dL)**	**260 (217, 420)**	**273 (229, 420)**	**247 (217, 360)**	**0.043**
Transferrin saturation (%)	25.5 (5.8, 49.0)	25.5 (5.8, 47.5)	24.0 (18.5, 49.0)	0.56
Ferritin (ng/mL)	34.5 (5.9, 381)	29.9 (5.9, 244)	48.1 (9.6, 381)	0.052
Hepcidin-25 (ng/mL)	17 (0.10, 141)	8.1 (0.10, 141)	22 (0.20, 135)	0.14
**Proportion of treatment of ESA (%)**	**84.6**	**77.8**	**100**	**0.027**
ERI (IU/week/g/dL/kg)	7.8 (2.6, 19)	8.2 (2.6, 19)	7.4 (4.4, 16)	0.76
Proportion of treatment of iron (%)	20.5	25.9	8.3	0.18
ECW/TBW	0.358 (0.331, 0.387)	0.358 (0.331, 0.372)	0.358 (0.333, 0.387)	0.50
**ICW**	**20.4 (15.4, 26.9)**	**21.4 ± 0.62**	**18.5 ± 0.97**	**0.017**

The bold text means significant results

*MCV* mean corpuscular volume, *BMI* body mass index, *DM* diabetes mellitus, *nPCR* normalized protein catabolic rate, *Hb* hemoglobin, *MCHC* mean corpuscular hemoglobin concentration, *ESA* erythropoiesis stimulating agents, *ERI* ESA resistance index, *ECW* extracellular water, *TBW* total body water, *ICW* intracellular water

^*^Median (ranges),

^†^Mean ± SD (ranges)

Table S1 shows the Spearman’s rank correlation analysis. MCV was significantly correlated with hepcidin-25 and ECW/TBW, whereas it was inversely correlated with TIBC, Cr, albumin and nPCR. TIBC was significantly correlated with Cr and ICW, and it was inversely correlated with hepcidin-25, ferritin, and ECW/TBW.

### Multivariate linear regression analysis for association with MCV and TIBC at baseline

Multivariate models including iron metabolism markers showed that MCV and hepcidin-25 were significantly associated with TIBC (Table 2).

**Table 2:** Multivariate linear regression analysis of biomarkers for TIBC

Dependent factor: TIBC									
	Univariate model			Multivariate models					
	Model 1			Model 2 (R^2^ = 0.57)			Model 3 (R^2^ = 0.56)		
	β	SE	p	β	SE	p	β	SE	p
MCV	**-5.1**	**1.1**	** < 0.0001**	**-2.6**	**1.2**	**0.037**	**-2.4**	**1.1**	**0.044**
Log hepcidin-25	**-19.6**	**3.7**	** < 0.0001**	**-13.5**	**5.7**	**0.025**	**-15.1**	**3.8**	**0.0006**
Log ferritin	**-30.7**	**7.4**	**0.0002**	-2.3	10.3	0.82			
albumin	62.0	31.6	0.059						
nPCR	83.1	50.1	0.11						
Cr	**8.1**	**3.2**	**0.016**	-1.8	3.9	0.64			
ICW	**6.6**	**2.9**	**0.028**	0.076	3.0	0.98	-0.43	3.0	0.89
ECW/TBW	**-2065**	**686**	**0.0050**	-1281	630	0.052			
Log CRP	-10.2	8.4	0.23						

The bold text means significant results

*TIBC* total iron binding capacity, *nPCR* normalized protein catabolic rate

Model 1 is a univariate model

Model 2 including mean corpuscular volume (MCV), log hepcidin-25, log ferritin, creatinine (Cr), intracellular water (ICW) and extracellular water (ECW)/total body water (TBW) as independent factors

Model 3 is including age, gender, MCV, log hepcidin-25, ICW and log erythropoiesis stimulating agent resistance index (ERI) as independent factors

### Change in iron status, nutritional markers, muscle mass markers, and ECW/TBW by MCV size

**Fig. 1:**
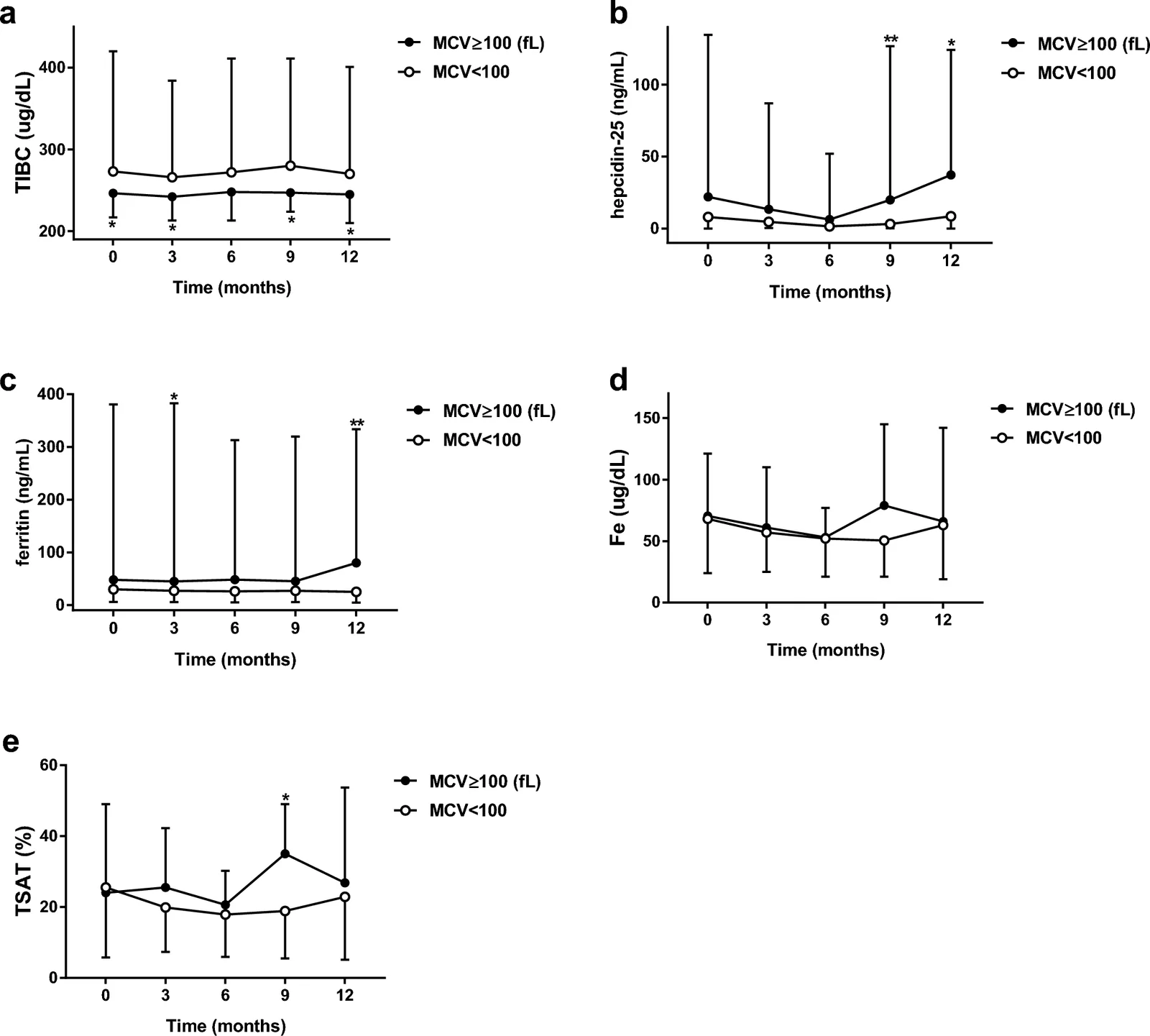
Changes in total iron binding capacity (TIBC) over 12 months between the patients in mean corpuscular volume (MCV) groups startified according to MCV size (**a**), changes in hepcidin-25 over 12 months between the patients in MCV groups (**b**), changes in ferritin over 12 months between the patients in MCV groups (**c**), changes in iron (Fe) over 12 months between the patients in MCV groups (**d**), changes in transferrin satration (TSAT) over 12 months between the patients in MCV groups (**e**) (p < 0.05*, p < 0.01**, p < 0.001***, p < 0.0001****)

**Fig. 2:**
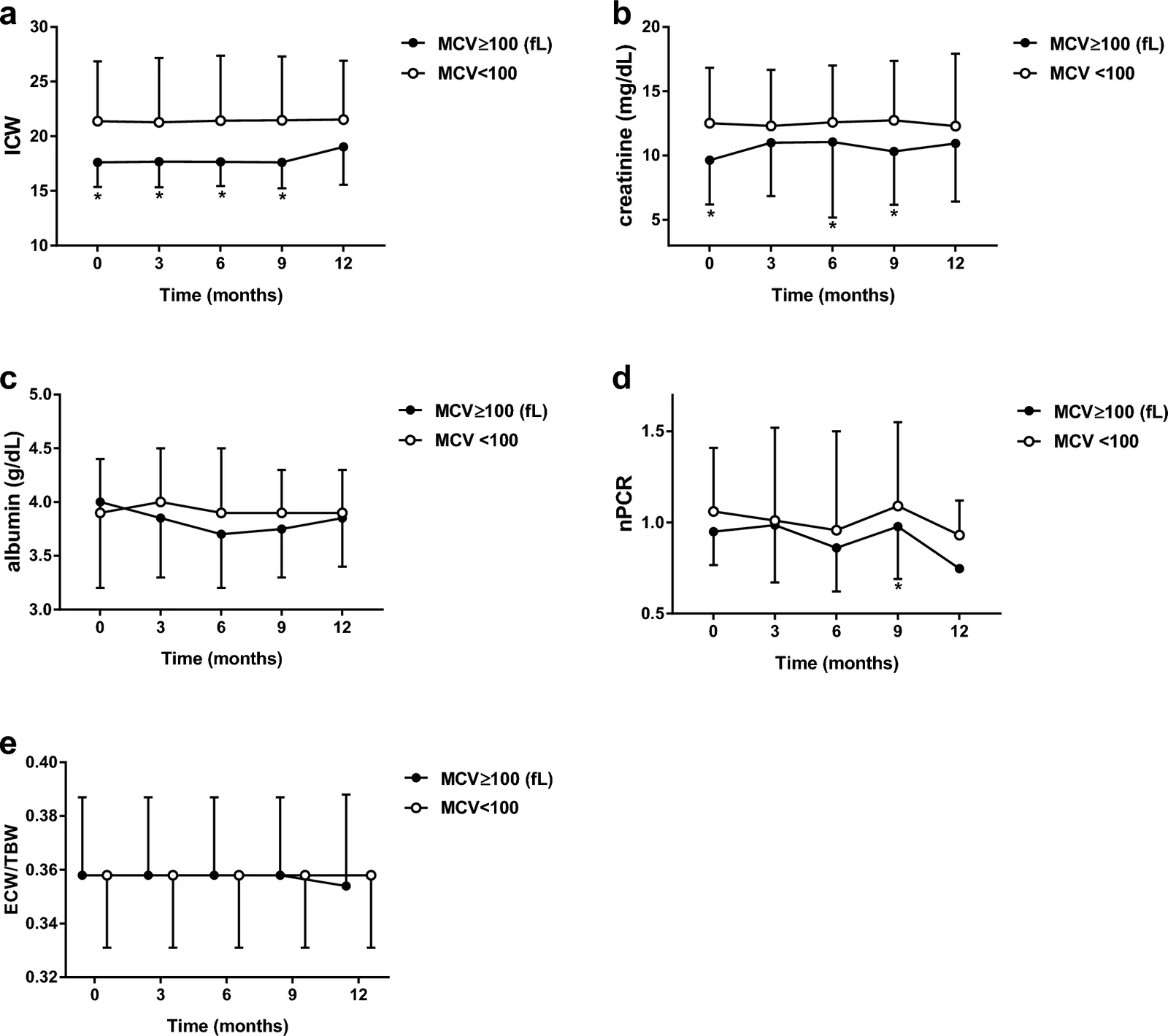
Changes in creatinine over 12 months between the patients in mean corpuscular volume (MCV) groups startified according to MCV size (**a**), changes in intracellular water (ICW) over 12 months between the patients in MCV groups (**b**), changes in albumin over 12 months between the patients in MCV groups (**c**), changes in normalized protein catabolic rate (nPCR) over 12 months between the patients in MCV groups (**d**), changes in extracellular body water/total body water ratio (ECW/TBW) over 12 months between the patients in MCV groups (**e**) (p < 0.05*, p < 0.01**, p < 0.001***, p < 0.0001****)

Figure 1 shows the change in iron status markers by MCV size. TIBC levels remained significant lower in the high MCV group. The median value of ferritin was changed from around 50 to around 80 for12 months in the same group. Hepcidin-25 levels were significantly higher from the 9th months. ICW was continuously lower in the high MCV group (Fig. 2). MCV remained significantly higher in the high MCV group (Fig. S1).

### Association between MCV and repeated measurements of TIBC by a multivariate approach

Table 3 shows the associations between MCV and repeated measurements of TIBC in a multivariate approach considering nutrition status, inflammation, muscle mass, and hydration status. MCV and hepcidin-25were significantly associated with TIBC. The interaction between hepcidin-25 and time points for TIBC was confirmed.

**Table 3:** Association between variables and repeated measurement TIBC by a multivariate approach

TIBC						
	Model 1		Model 2		Model 3	
	F	p	F	p	F	p
Timepoint	1.65	0.19	0.76	0.56	0.33	0.85
MCV	**18.7**	**0.0001**	**4.7**	**0.041**	**6.0**	**0.022**
Interaction of MCV and timepoint	1.44	0.25	0.60	0.67	0.55	0.70
Timepoint	**10.7**	** < 0.0001**				
Log hepcidin-25	**27.7**	** < 0.0001**	**5.5**	**0.28**	**4.7**	**0.041**
Interaction of loghepcidin-25 and timepoint	**6.08**	**0.0012**	2.7	0.065	**3.0**	**0.041**
Time	**3.48**	**0.020**				
Log ferritin	**17.1**	**0.0002**	0.021	0.89	0.34	0.56
Interaction of log ferritin and timepoint	**3.48**	**0.020**	2.5	0.079	2.5	0.073
Time	1.68	0.19				
Albumin	1.53	0.23				
Interaction of albumin and timepoint	1.71	0.18				
Time	0.049	0.99				
nPCR	1.10	0.30				
Interaction of nPCR and timepoint	0.20	0.94				
Time	1.13	0.36				
Cr	**4.76**	**0.037**	0.35	0.56	0.43	0.52
Interaction of Cr and timepoint	1.84	0.15	0.84	0.52	0.47	0.76
Time	0.98	0.44				
ICW	2.82	0.10				
Interaction of ICW and timepoint	1.31	0.29				
Time	0.93	0.46				
ECW/TBW	**5.84**	**0.023**	3.7	0.068		
Interaction of ECW/TBW and timepoint	0.87	0.50	0.69	0.61		
Time	**3.2**	**0.028**				
Log CRP	0.14	0.71	0.68	0.42		
Interaction of log CRP and timepoint	**2.80**	**0.045**	0.96	0.45		

The bold text means significant results

Model 1: Repeat measurement variables for total iron binding capacity (TIBC) was estimated with timepoint, each variable, interaction of timepoint and each variable

Model 2: Repeat measurement variables for TIBC was estimated with MCV, log hepcidin-25, log ferritin, creatinine (Cr), ECW/TBW (extracellular water/total body water), log C-reactive protein (CRP), timepoint, and interaction of timepoint

Model 3: Repeat measurement variables for TIBC was estimated with age, gender, MCV, log hepcidin-25, log ferritin, Cr, timepoint of each repeat measurement variable and interaction of timepoint

### Relationships among MCV, iron status, PEW and ERI

MCV was affected by hepcidin-25 and proportion of PEW and pre-PEW (Table S2). Table S3 shows that change in high MCV was affected by proportion of PEW and pre-PEW, and ERI. The interaction between proportion of PEW and pre-PEW, and time points for MCV was confirmed (models 2 and 3).

Table S4 showed that ERI was related to ICW in a multiple liner regression analysis.

## Discussion

This study demonstrated that a high MCV was associated with low TIBC in cross-sectional and longitudinal analyses considering nutritional status, inflammation, muscle mass, and hydration status. Furthermore, a sustained high MCV suggested ESA hypo-responsiveness, tendency to iron use disorder and risk of PEW in HD patients.

In terms of iron status, change in TIBC was significantly related to a high MCV. Hepcidin-25 was significantly higher in the high MCV group from the 9th month. A sustained high MCV may be affected by iron use disorder.

ERI was not related to high MCV at baseline, however, sustained high MCV was related to it. Erythropoiesis and iron homeostasis are reciprocally regulated and linked at multiple levels, with the erythroferrone (ERFE)-hepcidin-25 axis playing a major role in iron metabolism [21]. Produced and secreted by erythroblasts in response to erythropoietin, ERFE acts on the liver to suppress production of the iron regulatory hormone hepcidin-25 [22]. Bakr et al. reported that a high hepcidin-25/ERFE ratio suggests ESA hypo-responsiveness [21]. In this study, remained high MCV may reflect ESA hypo-responsiveness and tendency to iron use disorder [21, 23, 24]. ERI often has functional iron deficiency, however, levels of ferritin were not very high in this study. Some studies reported that the relationship among ERI, malnutrition and CRP. In this study, ERI was related to ICW in a multiple liner regression analysis. This result suggested that low muscle mass affected ERI to higher [25, 26].

In this study, muscle mass (Cr and ICW) was significantly lower with a high MCV. Change in muscle mass remained lower with a high MCV. High MCV was significantly related to proportion of PEW and pre-PEW. Although ERFE is also expressed in the skeletal muscle, its biological function and mechanism of regulation in the muscle remains to be clarified. Mina et al. reported that STAT3 activation in the skeletal muscle is a key signaling pathway that drives wasting and cachexia, also due to the high levels of IL-6 in cachectic patients and mice. They also reported that the BMP inhibitor ERFE is more expressed in models of cancer cachexia and in muscles of pre-cachectic and cachectic cancer patients [27]. Patients with FID need a higher ESA dose because the hematopoiesis mechanism does not work well due to a high hepcidin-25, and then the ERFE level becomes higher due to an increased ESA dose. This situation may cause muscle loss. A high MCV may suggest a higher ERFE because of iron use disorder; therefore, a high MCV was associated with muscle markers.

A high MCV was independently associated with a low TIBC, considering iron status markers, age, gender, nutritional status, inflammation, muscle mass, and hydration status on multivariate analysis.

TIBC is a maker of iron status and nutrition, and previous studies reported that a low TIBC was associated with muscle loss and FID [10]. TIBC is correlated with transferrin, which is reported to regenerate skeletal muscle [28, 29]. A high MCV was independently associated with TIBC, as well as hepcidin-25. The results of the present study demonstrated that a high MCV and a low TIBC suggest muscle loss and may reflect of risk of PEW.

There are some limitations in this study. First, the number of patients was small. Second, vitamin B12 and folic acid levels were not measured. Third, the treatment of anemia was managed by each physician at each clinic. We excluded patients with absolute iron deficiency according to the guideline for treatment of anemia from the Japanese Society of Dialysis Therapy [13]. However, Fer levels were not very high, and some patients are diagnosed absolute iron deficiency if we assess them according to the KDIGO 2026 Clinical Practice Guideline for the Management of Anemia in Chronic Kidney Disease [30], thus, there may be some patients who need more iron supply. Finally, the specific association between muscle mass and hydration status estimated by an anthropometric method such as bio-impedance spectroscopy (BIS) could not be assessed. Indeed, BIS and dual X-ray absorptiometry (DXA) are better ways to assess changes in muscle mass in dialysis patients. In the present study, body composition was not assessed by BIS or DXA. Muscle mass was estimated by Cr and ICW [18]. However, TBW was estimated by Watson’s formula; Arkouche et al. reported that Watson’s formula was the best of several ways including BIS to assess TBW [17]. In addition, in cases with tissue edema, estimation by BIS may be less reliable in patients on HD. To standardize measurements and avoid this kind of problem, the recent KDOQI guidelines on nutrition in CKD recommend that BIA should be performed at least 30 min after the HD session to allow for the distribution of body fluids [31, 32]. Based on these studies, using the formula can provide an approximate assessment.

In conclusion, this study demonstrated that a high MCV was associated with a low TIBC in cross-sectional and longitudinal analyses considering nutrition, inflammation, muscle mass, and hydration status. Furthermore, a sustained high MCV suggested ESA hypo-responsiveness and tendency to iron use disorder, and it may be useful markers of PEW.

## Supplementary Information

Below is the link to the electronic supplementary material.

Supplementary file1 (DOCX 21 KB)

Supplementary file2 (DOCX 23 KB)

Supplementary file3 (DOCX 27 KB)

Supplementary file4 (DOCX 22 KB)

Supplementary file5 (PDF 37 KB)
