# Supplementary material for: Relationships of high mean corpuscular volume, low total iron binding capacity and protein-energy wasting in patients on maintenance hemodialysis
Source: Clin Exp Nephrol. 2026 Jun 2;30(9):1350–8. doi: 10.1007/s10157-026-02887-w (PMC13518378; doi:10.1007/s10157-026-02887-w)
Supplement: Supplementary file 1 [file 10157_2026_2887_MOESM5_ESM.pdf]

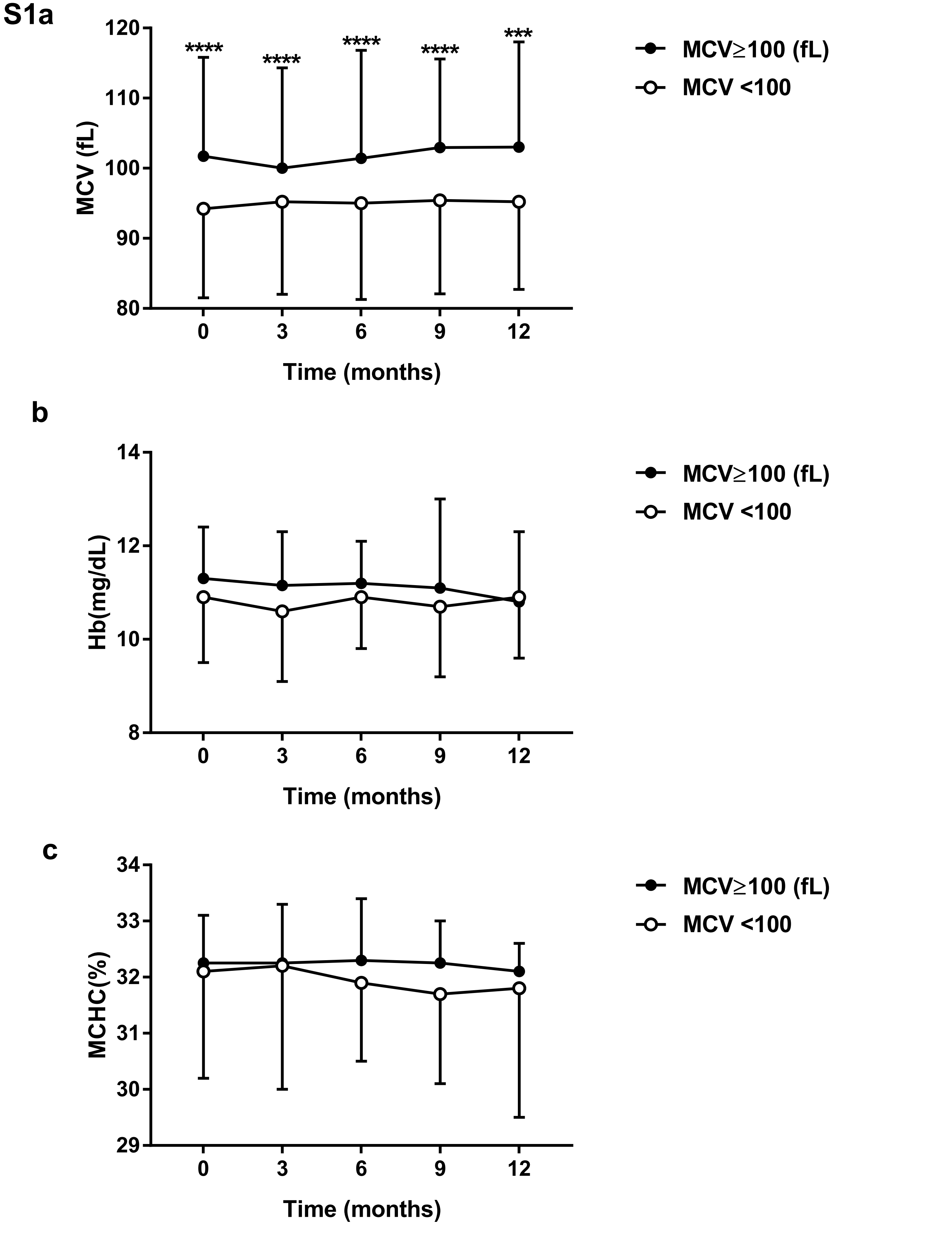

Figure S1. Changes in mean corpuscular volume (MCV) over 12 months between the patients in MCV groups startified according to MCV size (a), changes in hemoglobin (Hb) over 12 months between the patients in MCV groups(b), changes in mean corpuscular hemoglobin concentration (MCHC) over 12 months between the patients in MCV groups (c) ($p<0.05$*, $p<0.01$**, $p<0.001$***, $p<0.0001$****)
