# Supplementary material for: Relationships of high mean corpuscular volume, low total iron binding capacity and protein-energy wasting in patients on maintenance hemodialysis
Source: Clin Exp Nephrol. 2026 Jun 2;30(9):1350–8. doi: 10.1007/s10157-026-02887-w (PMC13518378; doi:10.1007/s10157-026-02887-w)
Supplement: Supplementary file 2 [file 10157_2026_2887_MOESM1_ESM.docx]

Table S1. Spearman’s rank correlation analysis at baseline

|  | MCV | TIBC | Ferritin | Hepcidin-25 | CRP | albumin | nPCR | Cr | ICW | ECW/TBW | ERI |
| --- | --- | --- | --- | --- | --- | --- | --- | --- | --- | --- | --- |
| age | 0.34^*^ | -0.47^**^ | 0.37^*^ | 0.12 | 0.19 | -0.59^***^ | -0.23 | -0.58^***^ | -0.41^*^ | 0.26^****^ | 0.22 |
| MCV |  | -0.61^****^ | 0.30 | 0.33^*^ | 0.24 | -0.29^*^ | -0.24^*^ | -0.41^*^ | -0.25 | 0.35^*^ | -0.030 |
| TIBC |  |  | -0.41^**^ | -0.44^**^ | -0.26 | 0.32 | 0.28 | 0.39^*^ | 0.38^*^ | -0.47^**^ | -0.16 |
| Ferritin |  |  |  | 0.74^****^ | -0.083 | 0.017 | -0.29 | -0.48^**^ | -0.40^*^ | 0.40^*^ | 0.053 |
| Hepcidin-25 |  |  |  |  | 0.010 | 0.31 | 0.013 | -0.35^*^ | -0.36^*^ | 0.20 | -0.070 |
| CRP |  |  |  |  |  | -0.15 | -0.078 | -0.21 | -0.028 | 0.26 | 0.16 |
| albumin |  |  |  |  |  |  | 0.51^**^ | 0.21 | 0.26 | -0.58^***^ | -0.36 |
| nPCR |  |  |  |  |  |  |  | 0.14 | 0.074 | -0.27 | -0.25 |
| Cr |  |  |  |  |  |  |  |  | 0.70^****^ | -0.54^**^ | -0.39^*^ |
| ICW |  |  |  |  |  |  |  |  |  | -0.48^**^ | -0.57 |
| ECW/TBW |  |  |  |  |  |  |  |  |  |  | 0.16 |

*: p<0.05, **: p<0.01, ***: p<0.001, ****: p<0.0001

MCV: mean corpuscular volume, TIBC: total iron binding capacity, CRP: C-reactive protein, nPCR: normalized protein catabolic rate, Cr: creatinine, ICW: intracellular water, ECW: extracellular water, TBW: total body water, ERI: erythropoiesis stimulating agent resistance index
