# Supplementary material for: Relationships of high mean corpuscular volume, low total iron binding capacity and protein-energy wasting in patients on maintenance hemodialysis
Source: Clin Exp Nephrol. 2026 Jun 2;30(9):1350–8. doi: 10.1007/s10157-026-02887-w (PMC13518378; doi:10.1007/s10157-026-02887-w)
Supplement: Supplementary file 3 [file 10157_2026_2887_MOESM2_ESM.docx]

Table S2. Multivariate linear regression analysis of biomarkers for MCV

| **Dependent factor: MCV** | | | | | | | | | |  |
| --- | --- | --- | --- | --- | --- | --- | --- | --- | --- | --- |
|  | Univariate model | | | Multivariate models | | | | | |  |
|  | Model 1 | | | Model 2 (R^2^= 0.45) | | | Model 3 (R^2^= 0.40) | | | |
|  | β | SE | p | β | SE | p | β | SE | p | |
| Log hepcidin-25 | **1.6** | **0.52** | **0.0038** | **1.4** | **0.49** | **0.59** | **1.5** | **0.49** | **0.0064** | |
| Log ferritin | 2.0 | 1.0 | 0.050 |  |  |  |  |  |  | |
| albumin | -6.6 | 3.8 | 0.090 |  |  |  |  |  |  | |
| nPCR | **-13.3** | **5.7** | **0.027** | -6.1 | 6.2 | 0.33 |  |  |  | |
| Cr | **-1.0** | **0.39** | **0.012** | -0.26 | 0.48 | 0.59 |  |  |  | |
| ICW | -0.52 | 0.36 | 0.16 |  |  |  |  |  |  | |
| ECW/TBW | **183** | **86.1** | **0.041** | 14.7 | 84.1 | 0.86 |  |  |  | |
| Log CRP | 1.2 | 0.99 | 0.23 |  |  |  |  |  |  | |
| Proportion of PEW and pre-PEW | **5.2** | **1.6** | **0.0025** | **5.4** | **1.9** | **0.0091** | **6.3** | **1.6** | **0.0007** | |
| Log ERI | 0.18 | 2.6 | 0.95 | **-5.2** | **2.3** | **0.037** | -4.4 | 2.2 | 0.062 | |

MCV: mean corpuscular volume, ICW: intracellular water

Model 1 is a univariate model.

Model 2 including log hepcidin-25, normalized protein catabolic rate (nPCR), creatinine (Cr), extracellular water/total body water (ECW/TBW), proportion of protein-energy wasting (PEW) and pre-PEW and log erythropoiesis stimulating agent resistance index (ERI) as independent factors.

Model 3 is including age, gender, log hepcidin-25, proportion of PEW and pre-PEW and log ERI as independent factors.
