# Supplementary material for: Relationships of high mean corpuscular volume, low total iron binding capacity and protein-energy wasting in patients on maintenance hemodialysis
Source: Clin Exp Nephrol. 2026 Jun 2;30(9):1350–8. doi: 10.1007/s10157-026-02887-w (PMC13518378; doi:10.1007/s10157-026-02887-w)
Supplement: Supplementary file 4 [file 10157_2026_2887_MOESM3_ESM.docx]

Table S3. Association between variables and repeated measurement MCV by a multivariate approach

| MCV | | | | | | | | | |
| --- | --- | --- | --- | --- | --- | --- | --- | --- | --- |
|  | Model 1 | | Model 2 | | | Model 3 | | | |
|  | F | p | F | p | | F | | p | |
| timepoint | **4.1** | **0.011** | **3.1** | | **0.045** | | 1.1 | | 0.38 |
| Log hepcidin-25 | **4.4** | **0.046** | 4.1 | | 0.058 | | 3.4 | | 0.082 |
| Interaction of loghepcidin-25 and timepoint | 0.32 | 0.86 | 0.88 | | 0.50 | | 0.75 | | 0.57 |
| time | 0.95 | 0.45 |  | |  | |  | |  |
| Log ferritin | 3.6 | 0.068 |  | |  | |  | |  |
| Interaction of log ferritin and timepoint | 0.77 | 0.55 |  | |  | |  | |  |
| time | 2.6 | 0.062 |  | |  | |  | |  |
| albumin | 1.2 | 0.28 |  | |  | |  | |  |
| Interaction of albumin and timepoint | **3.2** | **0.033** |  | |  | |  | |  |
| time | **4.8** | **0.0059** |  | |  | |  | |  |
| nPCR | 2.7 | 0.11 | 1.1 | | 0.31 | |  | |  |
| Interaction of nPCR and timepoint | **3.2** | **0.032** | 2.2 | | 0.12 | |  | |  |
| time | 1.0 | 0.42 |  | |  | |  | |  |
| Cr | 3.7 | 0.064 | 0.58 | | 0.46 | | 0.44 | | 0.52 |
| Interaction of Cr and timepoint | 1.6 | 0.21 | 0.86 | | 0.51 | | 0.89 | | 0.49 |
| time | 0.80 | 0.54 |  | |  | |  | |  |
| ICW | 0.88 | 0.36 |  | |  | |  | |  |
| Interaction of ICW and timepoint | 1.6 | 0.21 |  | |  | |  | |  |
| time | 1.4 | 0.26 |  | |  | |  | |  |
| ECW/TBW | 3.6 | 0.071 |  | |  | |  | |  |
| Interaction of ECW/TBW and timepoint | 1.2 | 0.33 |  | |  | |  | |  |
| time | 1.5 | 0.24 |  | |  | |  | |  |
| Log CRP | 0.047 | 0.83 |  | |  | |  | |  |
| Interaction of log CRP and timepoint | 1.4 | 0.26 |  | |  | |  | |  |
| time | **4.0** | **0.013** |  | |  | |  | |  |
| Proportion of PEW and pre-PEW | **6.7** | **0.015** | **5.7** | | **0.028** | | **9.4** | | **0.0066** |
| Interaction of PEW and pre-PEW and timepoint | **6.3** | **0.0015** | **3.8** | | **0.024** | | **3.1** | | **0.047** |
| time |  |  |  | |  | |  | |  |
| Log ERI | 0.56 | 0.46 | **4.6** | | **0.046** | | **5.1** | | **0.037** |
| Interaction of log ERI and timepoint | 1.3 | 0.30 | 0.77 | | 0.56 | | 0.97 | | 0.45 |

Model 1: Repeat measurement variables for mean corpuscular volume (MCV) was estimated with timepoint, each variable, interaction of timepoint and each variable.

Model 2: Repeat measurement variables for MCV was estimated with log hepcidin-25, normalized protein catabolic rate (nPCR), creatinine (Cr), proportion of protein-energy wasting (PEW) and pre-PEW, log erythropoiesis stimulating agent resistance index (ERI), timepoint, and interaction of timepoint.

Model 3: Repeat measurement variables for MCV was estimated with age, gender, log hepcidin-25, Cr, proportion of PEW and pre-PEW, log ERI, timepoint, and interaction of timepoint.
