# Supplementary material for: Relationships of high mean corpuscular volume, low total iron binding capacity and protein-energy wasting in patients on maintenance hemodialysis
Source: Clin Exp Nephrol. 2026 Jun 2;30(9):1350–8. doi: 10.1007/s10157-026-02887-w (PMC13518378; doi:10.1007/s10157-026-02887-w)
Supplement: Supplementary file 5 [file 10157_2026_2887_MOESM4_ESM.docx]

Table S4. Multivariate linear regression analysis of biomarkers for ERI

| **Dependent factor: ERI** | | | | | | | | | |  |
| --- | --- | --- | --- | --- | --- | --- | --- | --- | --- | --- |
|  | Univariate model | | | Multivariate models | | | | | |  |
|  | Model 1 | | | Model 2 (R^2^= 0.36) | | | Model 3 (R^2^= 0.37) | | | |
|  | β | SE | p | β | SE | p | β | SE | p | |
| Log hepcidin-25 | 0.033 | 0.045 | 0.47 | 0.0063 | 0.060 | 0.92 | 0.0066 | 0.042 | 0.88 | |
| Log ferritin | 0.00036 | 0.0011 | 0.73 | -0.020 | 0.11 | 0.86 |  |  |  | |
| albumin | -0.53 | 0.30 | 0.088 | -0.54 | 0.36 | 0.15 | -0.70 | 0.34 | 0.050 | |
| nPCR | -0.49 | 0.48 | 0.31 | -0.20 | 0.49 | 0.68 |  |  |  | |
| Cr | **-0.087** | **0.031** | **0.0094** |  |  |  |  |  |  | |
| ICW | **-0.093** | **0.023** | **0.0004** | **-0.083** | **0.026** | **0.0039** | **-0.087** | **0.027** | **0.0033** | |
| ECW/TBW | 7.2 | 6.8 | 0.30 |  |  |  |  |  |  | |
| Log CRP | -0.043 | 0.10 | 0.68 | -0.20 | 0.11 | 0.092 | -0.19 | 0.12 | 0.13 | |

ERI: erythropoiesis stimulating agent resistance index

Model 1 is a univariate model.

Model 2 including log hepcidin-25 and log ferritin, normalized protein catabolic rate (nPCR), creatinine (Cr) and extracellular water/total body water (ECW/TBW) as independent factors.

Model 3 is including age, gender, log hepcidin-25, albumin, ICW and log C-reactive protein (CRP) as independent factors.
